# Development and Functions of the Infant Gut Microflora: Western *vs*. Indian Infants

**DOI:** 10.1155/2020/7586264

**Published:** 2020-05-07

**Authors:** Lalit Bharadia, Neha Agrawal, Nandan Joshi

**Affiliations:** ^1^Santokba Durlabhji Memorial Hospital cum Medical Research Institute, Bhawani Singh Marg, Jaipur, 302015 Rajasthan, India; ^2^Danone India, Phoenix Market City, Centrium Gate 1, LBS Road, Kurla (West), Mumbai, 400070 Maharashtra, India

## Abstract

The human gut is colonized by trillions of bacteria as well as other microorganisms, collectively referred to as the “gut microflora.” This microflora plays an important role in metabolism as well as immunity, and alterations in its normal composition and pattern of colonization can disturb the development and functioning of the immune system, predisposing the individual to several diseases. Neonates acquire their gut microflora from the mother as well as the surroundings, and as the infant grows, the gut microflora undergoes several changes, ultimately acquiring an adult-like composition. Characterization of the gut microflora of healthy infants is important to protect infants from infectious diseases. Furthermore, formulation of prebiotics and probiotics for boosting infant immunity in a specific population also requires prior knowledge of the normal gut microflora in a healthy infant in that population. To this end, several studies have been performed on Western infants; however, the gut microflora of Indian infants is as yet insufficiently studied. Moreover, there has been no comparative analysis of the development and characteristics of the infant gut microflora between the two populations. In this review, we discuss the development and maturation of the infant gut microflora and its effect on immunity, as well as the factors affecting the patterns of colonization. In addition, we compare the patterns of colonization of gut microflora between Western and Indian infants based on the available literature in an attempt to identify the extent of similarity or difference between the two populations.

## 1. Introduction

The human intestinal tract is home to a complex microbial ecosystem comprising approximately 100 trillion bacteria belonging to over 35,000 species, as well as other microorganisms such as fungi, archaea, viruses, and protozoans [1, 2]. The gut microflora plays an important role in maintaining the health of the gut as well as that of the entire individual [2]. A number of these bacteria, such as lactobacilli and bifidobacteria, have been shown to be involved in the development and functioning of the immune system [3, 4], resistance to infections by preventing excessive growth of pathogenic microbes [5, 6], nutrient metabolism [7], drug metabolism, intestinal barrier function, detoxification of xenobiotic compounds, and activation of compounds beneficial for the human health (e.g., polyphenols) [1, 2]. The composition of the gut microflora in infants is different from that in adults. The first microbes to colonize the neonatal gut are instrumental in establishing the infant gut microflora. They have a major impact on its long-term composition and activity, which are key determinants of the individual's overall health and immunity later in life. Any changes in the normal pattern of colonization at this stage can result in gut microflora dysbiosis throughout adult life, thus predisposing the individual to diseases. Hence, establishment of a healthy gut microflora during the early (typically, the first 2) years of life is extremely important [1].

The development and characteristics of the infant gut microflora in the Western world have been studied and reviewed extensively; however, few such studies have been conducted on Indian infants. Moreover, there is also a lack of studies comparing the gut microflora between Western and Indian infants. In this review, we discuss the development of the infant gut microflora, its contribution to immunity, and the various factors affecting its composition. We also perform a comparative analysis of the available literature on the gut microflora of Western and Indian infants to identify if there are any similarities in the pattern of colonization between the two populations.

### 1.1. Search Strategy

PubMed was searched for relevant articles by using the following keywords and phrases: “infant gut microflora,” “infant gut microbiota,” “infant intestinal microflora,” and “infant intestinal microbiota.” These phrases were combined with the term “India” to specifically search for studies on Indian infants. The full texts of the retrieved articles as well as those of relevant articles from the bibliographic lists of these articles were reviewed.

## 2. Results

### 2.1. Development of the Infant Gut Microflora

For a long time, it was believed that a newborn infant has an essentially sterile gut, which starts getting colonized by microorganisms during and immediately after delivery [1]. However, some recent studies have challenged this long-held belief by demonstrating the presence of bacteria in the umbilical cord blood [8], amniotic fluid [9], and placenta [10] in healthy pregnancies, indicating that exposure of the fetus to microbes may start even before delivery. As of now, there are conflicting views on this issue, with some researchers supporting the “sterile womb” view [11, 12] and others opposing it [8–10].

Immediately after birth, the infant is exposed to the extrauterine environment with high levels of live microbes, and rapid colonization of the neonatal intestine takes place [1]. During and shortly after birth, the mother, particularly the maternal gut microflora, is the primary source of microbes for the neonatal gut [13]. The first microbes to colonize the neonatal gut are facultative anaerobes. They use up the oxygen within the gut and pave the way for the growth of a complex microbial community predominantly composed of obligate anaerobes [14]. Mother's milk is usually an infant's first diet and its sole source of nutrition for around 6 months after birth. It contains nondigestible carbohydrates that are fermented in the colon and stimulate the growth of specific fecal bacteria [15]. In addition to this “prebiotic effect,” the mother's milk is also a source of live bacteria, including staphylococci, streptococci, bifidobacteria, and lactic acid bacteria [16–18]. The origin of these bacteria remains controversial. One view is that the mother's fecal microbiota is transferred to the infant during delivery, from where it gets transferred to the breast skin and nipple and ultimately to the milk ducts while breastfeeding [1]. It is also believed that some of the maternal gut microbiota can reach the mammary glands *via* an endogenous route [19].

At the age of 4-6 months, infants in industrialized countries are generally introduced to solid foods, such as cereals, fruits, and vegetables. The new types of nondigestible carbohydrates specific to these foods result in a major change in the composition of the intestinal microflora [1]. The intestinal microflora of children shows greater interindividual variability than that of adults; however, with the passage of time, these interindividual differences decrease. Concomitantly, the species diversity of the infant gut microflora increases as it becomes more complex and begins to resemble the gut microflora of adults [20–22]. Generally, by the age of 2.5-3 years, a stable, adult-like gut microbiota is established [20, 22].

### 2.2. Factors Affecting the Development of Infant Gut Microflora

Several factors influence the growth and establishment of the infant gut microflora. Both the gestational age at birth and mode of delivery (vaginal *vs*. cesarean) affect the composition of the neonatal gut microflora in the initial period after birth [1]. Premature birth has been found to be associated with delayed colonization with a limited number of species [23, 24]. This difference has been attributed to the aseptic environment of the neonatal intensive care unit and the extensive use of antibiotics shortly after birth in preterm infants [1]. Similarly, cesarean delivery also leads to delayed colonization (especially by bifidobacteria) and lower diversity of gut microflora compared with vaginal delivery [25]. While vaginally born infants acquire their initial gut microflora mainly from the maternal vaginal and perineal microbiota, in case of cesarean-born infants, the main contributors are the mother's skin and the nosocomial environment [1]. In a study comparing the gut microflora of vaginally born and cesarean-born infants, it was found that in vaginally born infants, the composition of the gut microflora resembled that of the mothers' vaginal microflora, including mainly *Lactobacillus*, *Prevotella*, and *Sneathia* spp. In contrast, the microflora of cesarean-born infants was similar to that found on the mothers' skin surface and was dominated by *Staphylococcus*, *Corynebacterium*, and *Propionibacterium* spp. [26]. It has also been shown that the disturbances in the gut microflora of cesarean-born infants may persist up to the age of 6 months [27].

As the infant grows, its environment (family lifestyle and geographical location) also influences the development of the gut microflora. Infants in developed Western countries are brought up in a more hygienic environment that limits their exposure to pathogenic microbes. In contrast, infants growing up in developing countries have a greater bacterial exposure. This affects the gut colonization process [1]. Few studies have made direct comparisons between the gut microflora of infants in developed and developing countries; however, it is well known that colonization with enterobacteria such as *Escherichia coli* is delayed in infants from developed countries compared with those in developing countries. The former are colonized by fewer bacterial strains, with a lower turnover rate than that observed in infants from developing countries [28, 29]. Even in the developed world, geographical as well as ethnic differences have been shown to affect the colonization patterns of gut microflora in infants [30, 31].

Last but not the least, the mode of feeding also has a considerable influence on the development of infant gut microflora. Breast milk provides a mixture of specific nutrients and pro- and antimicrobial agents, which lend characteristic features to the gut microbiota in breastfed infants. The presence of human milk oligosaccharides in breast milk facilitates the growth and functioning of beneficial microbes [14]. It is a well-established fact that breastfed and formula-fed infants have distinct gut microflora; the gut microflora of exclusively breastfed infants has higher proportions of Actinobacteria (particularly bifidobacteria), which is a protective bacterial class. This was demonstrated as early as 1981 by Daoulas Le Bourdelles et al., who compared the fecal microflora of a group of breastfed French neonates with that of another group of bottlefed neonates [32]. Subsequently, several other researchers also reported the same observation [20, 33–36]. Moreover, the gut microflora of formula-fed infants is more diverse than that of breastfed infants and acquires adult-like characteristics earlier than the latter [21, 33, 34].

### 2.3. Effect of Infant Gut Microflora on the Immune System

The intestinal microflora plays an important role in the maturation and functioning of the immune system. Alterations in the normal composition of the gut microflora can lead to immune dysregulation and predispose the child to immune-related disorders, such as allergy, obesity, or diabetes, later in life [1]. Some of the immune-related functions of the intestinal microflora are discussed in the following sections.

#### 2.3.1. Development and Modulation of the Intestinal Immune System

The gut microbiota plays a crucial role in the development of the gut-associated lymphoid tissue, which is an important player in the defense against microorganisms and environmental antigens [1]. It has been shown that gut lymphocytes undergo important developmental changes after coming in contact with intestinal antigens in local lymphoid structures, such as Peyer's patches. Studies in germ-free mice have revealed important roles of intestinal bacteria in the maturation and functioning of the mucosal immune system, for example, stimulating the production of mucosal immunoglobulin A (IgA) and contributing to the development of intraepithelial lymphocytes [37]. DNA microanalyses have shown that *Bacteroides thetaiotaomicron*, upon colonizing germ-free mice, modulates the expression of host genes involved in postnatal maturation, nutrient uptake and metabolism, xenobiotic processing, and angiogenesis [38]. Mazmanian et al. showed that maturation of the mammalian immune system is directed by a specific immunomodulatory polysaccharide, polysaccharide A (PSA), provided by *Bacteroides fragilis*, a ubiquitous gut microorganism. The immunomodulatory effects of PSA include correction of systemic T cell deficiencies and T_h_1/T_h_2 imbalances and directing lymphoid organogenesis [39].

It has also been shown that intestinal microbes regulate the development of the intestinal villus microvasculature through Paneth cells [40]. Intestinal bacteria also play important roles in the development of specific lymphocytes; they trigger class switching in human intestinal B cells [41] and regulate the development of T_h_17 effector T cells in the mucosa of the small intestine [42]. They also suppress the production of Treg cells in specific situations where it is necessary to limit the Treg level to ensure an efficient immune response, for example, in case of oral vaccination or to control acute infections [43]. Gut microbes can also modify the immunomodulatory properties of native food proteins. It was found that bovine casein degraded by *Lactobacillus rhamnosus* GG, a gut microbe commonly used as a probiotic, suppressed interleukin-4 (IL-4) production by anti-CD3 antibody-induced peripheral blood mononuclear cells obtained from infants with atopic disease [44]. In line with this observation, a landmark trial by Kalliomäki et al. later showed *Lactobacillus rhamnosus* GG to be effective in the prevention of atopic disease in high-risk infants (i.e., those who had a family history of atopic disease) [45].

#### 2.3.2. Establishing and Regulating the Intestinal Surface Barrier

Several studies have shown that the intestinal microflora maintains and fortifies the intestinal mucosal barrier [46–49]. Cani et al. showed that prebiotic modulation of the intestinal microbiota resulting in a selective increase in *Bifidobacterium* spp. lowers intestinal permeability and improves tight junction integrity in obese mice [47]. Commensal bacteria have also been shown to play a role in the maintenance of intestinal epithelial homeostasis and protection of the intestinal epithelium from damage. This function of gut commensals is mediated *via* the activation of intestinal Toll-like receptors [48]. Furthermore, in another study, a soluble protein, p40, derived from *Lactobacillus rhamnosus* GG was shown to inhibit cytokine-induced apoptosis of intestinal epithelial cells both in vitro and in vivo [49]. Several gut bacteria, such as bifidobacteria, produce short-chain fatty acids (SCFAs; e.g., acetate) by fermentation of nondigestible carbohydrates in the anaerobic gut lumen [50]. These SCFAs fortify the mucus layer covering the intestinal epithelium, thereby enhancing the gut barrier function and preventing adherence and colonization of pathogenic microbes [51]. Gut commensals also regulate the production of secretory IgA, an important component of mucosal immunity that prevents the adhesion of pathogenic microbes to the intestinal epithelium [52].

#### 2.3.3. Colonization Resistance and Pathogen Clearance

There is ample evidence suggesting that the gut microbiota plays a major role in colonization resistance, i.e., preventing the colonization of the intestine by entering pathogens and inhibiting the overgrowth of potentially pathogenic bacteria that are naturally present in the intestine in low numbers [1, 36]. Gut commensals employ various mechanisms to offer colonization resistance, including (a) production of antimicrobial substances such as bacteriocins (microbiocidal compounds for killing competitor strains), (b) modification of the pH of the luminal content, and (c) competition for nutrients required by the pathogenic microbes [53].

Gut commensals have been shown to play another role in providing protection against pathogenic microbes, the so-called pathogen clearance, wherein commensals eliminate pathogenic microbes from the gut lumen after an acute infectious episode (e.g., after *Salmonella* diarrhea). The difference between colonization resistance and pathogen clearance is that in the former, gut microbiota prevent the growth of pathogenic microbes right at the outset, whereas in the latter, the gut microbiota regrows from a state of depletion and gradually eliminates very high pathogen loads from the intestine. Pathogen clearance is believed to be mediated by some of the same mechanisms that are involved in colonization resistance; in addition, stimulation of the mucosal cellular immune system by the gut microbiota may also be involved [54].

### 2.4. Patterns of Colonization of Gut Microflora in Infants: Western *vs*. Indian Infants

There have been several studies on the sequential process of colonization of gut microflora in infants in Western countries. Based on these studies, the development of gut microbiota in infants has been divided into four separate phases: the first 1-2 weeks after birth, i.e., the initial phase of bacterial acquisition (Phase 1), the remaining period during which the infant is exclusively breastfed (Phase 2), the time between the beginning of supplementation and discontinuation of breastfeeding (Phase 3), and the period after completion of weaning when the infant gut microflora begins to resemble that of adults (Phase 4) [55].

The neonatal gut microflora comprises four major phyla: Proteobacteria, Firmicutes, Actinobacteria, and Bacteroidetes [14]. As mentioned previously, it has been established that the first microbes to colonize the infant gut are facultative anaerobes, such as *E*. *coli* and *Streptococcus* [55]. These are followed by *Staphylococcus*-, *Enterococcus*-, and *Lactobacillus*-like species, which use up the oxygen in the gut to create an anaerobic environment that would facilitate the growth of strictly anaerobic bacteria [1]. By postnatal day 4-7, anaerobic bacteria belonging to the genera *Bacteroides*, *Bifidobacterium*, and *Clostridium* colonize the gut [36, 55, 56]. During the next phase when the infant is exclusively breastfed, bifidobacteria predominate the gut microflora, while the proportions of *E*. *coli*, *Streptococcus*, *Bacteroides*, and *Clostridium* decrease [55].

With the introduction of solid foods at the age of 4-6 months, the composition of the infant gut flora undergoes further change. In a study by Stark and Lee, the introduction of solid weaning foods to breastfed infants resulted in a rapid increase in the number of enterobacteria and enterococci, after which progressive colonization by *Bacteroides* spp., clostridia, and anaerobic streptococci occurred. Colonization by bifidobacteria continued throughout the first year of life [57]. More recently, Amarri et al. obtained similar results in a study in which infants who were exclusively breastfed for around 4 months before the introduction of complementary feeding were followed up from 4 to 9 months of age. Counts of bifidobacteria were found to remain high throughout the 5 months of complementary feeding, while the counts of enterobacteria and enterococci increased with age. Counts of lactobacilli and vancomycin-insensitive lactobacilli increased from 4 to 7 months of age and then decreased [58]. By around the second year of life, the infant gut microbiota begins to resemble that of adults, with the presence of many other microbial groups, such as eubacteria, veillonella, staphylococci, propionibacteria, bacilli, fusobacteria, and yeasts [55]. The evolution of the gut microbiota in Western breastfed infants has been summarized in Table 1.

There have been relatively fewer studies on the development of gut microflora in Indian infants. However, as mentioned previously, it is well established that geographical and ethnic differences result in differences in the pattern of colonization of intestinal microflora in infants [28–31]. Hence, some differences in the gut microflora between Indian and Western infants are expected. Albert et al. performed one of the earliest studies on the gut microflora in Indian infants. In their study, they found the normal fecal flora of south Indian infants aged 1-20 months to be predominantly anaerobic, with bifidobacteria being the most abundant microbes. The aerobic flora primarily comprised enterobacteria and enterococci [59]. Similar results were obtained more recently in a study by Balamurugan et al. in which enterobacteria (*E*. *coli*) and bifidobacteria (*Bifidobacterium longum* subspecies *infantis*) were found to be the dominant fecal bacteria in a cohort of term-born neonates in a tertiary care hospital in southern India [60]. In another study conducted in southern India, bifidobacteria were found to be prominent members of the fecal flora in children aged 2-3 years, and *Lactobacillus acidophilus* was also detected. However, both bacteria decreased in abundance after this age [61]. All these studies had small sample sizes, with the number of infants not exceeding 30. Kabeerdoss et al. performed a study with a larger sample size, in which 83 term-born infants in a South Indian hospital were followed up from birth till 6 months of age. The results obtained were largely similar to those of the aforementioned studies: enterobacteria and lactobacilli were the predominant gut bacteria in the first 2 days of life, whereas bifidobacteria and staphylococci increased by the fourth day. The *Bacteroides-Prevotella* group, a major component of the adult gut microbiota, was relatively less abundant after birth but increased by around 6 months of age. Enterococci were less abundant in these infants [62].

In a study performed in Maharashtra, India, Pandey et al. found the fecal microflora of full-term, vaginally born, breastfed infants on postnatal day 7 to be dominated by *Acinetobacter* sp. (accounting for 44% of the fecal microflora), followed by *Bifidobacterium* sp. and *Staphylococcus* sp. [63]. This abundance of bifidobacteria on postnatal day 7 was in line with the observations of previous studies in Western infants [55]. In contrast, the fecal microflora of cesarean-born infants was dominated by *Citrobacter* sp., *E. coli*, and *Clostridium difficile.* Interestingly, bifidobacteria were absent in these infants, despite the fact that they were also exclusively breastfed [63]. Dinh et al. followed persistently stunted and normal children (controls) from a birth cohort in a South Indian slum community from the age of 3 months to 2 years and compared their intestinal microbiota. They found that the most abundant phyla in the overall cohort were Firmicutes (38.6%) and Proteobacteria (25.89%), followed by Actinobacteria (17.5%), Bacteroidetes (13.8%), and Verrucomicrobia (2.6%) [64]. This pattern of colonization was similar to those observed in previous studies on infants in the United States [22, 65] and Italy [66], where Proteobacteria and Firmicutes were found to be the predominant phyla in the intestinal microflora. It was also observed that the microflora of control children was enriched in the probiotic species *Bifidobacterium longum* and *Lactobacillus mucosae*, which is in line with their health benefits.

Chandel et al. analyzed the intestinal microflora of full-term infants delivered by cesarean section in a hospital in Odisha, India. On postnatal day 7, Firmicutes (28%; *Enterococcus, Clostridium, Staphylococcus*) and Proteobacteria (64%; *Escherichia, Shigella*) were the predominant phyla. However, by day 60, the proportion of Proteobacteria had reduced significantly to 13%, and Firmicutes (49%; *Streptococcus*), Bacteroidetes (36%), and Actinobacteria (*Bifidobacterium*) had increased in abundance [67]. The pattern of colonization on day 60 in this study was different from that observed in a US study by Palmer et al., in which a higher proportion of Proteobacteria (46%) and lower proportions of Firmicutes (32%) and Bacteroidetes (20%) were observed [65].

Most of the abovementioned studies had been performed in southern India. Hence, it was important to analyze the intestinal microflora in North Indian infants as well. Attri et al. analyzed the intestinal microflora of vaginally born, exclusively breastfed, full-term infants in Solan and Shimla, Himachal Pradesh. In the first and second months of life, facultative anaerobic bacteria from the phyla Firmicutes and Proteobacteria predominated, while in the third and fourth months, an increase in obligate anaerobes from the phyla Bacteroidetes and Actinobacteria was observed [68]. This pattern of succession was similar to that observed in the previous study by Chandel et al. involving infants in Odisha [67], indicating that the gut microflora of infants from different parts of India may not be so different after all. In the study by Attri et al., Clostridia remained predominant throughout the first 4 months of life (with a relative abundance of 20-30%). The *Bifidobacterium* species detected included *Bifidobacterium adolescentis*, *B. bifidum*, *B. longum*, *B. pseudolongum*, and *B. breve* [68]. In another study, members of the same group analyzed the establishment and diversity of lactic acid-producing bacteria and bifidobacteria among full-term, vaginally born, exclusively breastfed infants from the same geographic area for the first 4 months of life. They found that among the lactic acid-producing bacteria, species of the genera *Enterococcus*, *Streptococcus*, and *Lactobacillus* were predominant, whereas among bifidobacteria, *B. breve* was the predominant species. The diversity of both lactic acid-producing bacteria and bifidobacteria increased with time over the 4 months of the study [69]. In a study performed in one of the leading hospitals of New Delhi, India, analysis of the fecal microflora of 29 full-term infants around 4 weeks of age revealed the presence of strictly aerobic, facultatively anaerobic, and anaerobic bacteria. The predominant aerobic organisms were *Micrococcus* sp. (3.4%); facultative anaerobes included *Klebsiella pneumoniae* (41.3%), *E. coli* (24.1%), *Proteus* sp. (10.3%), *Enterococcus faecium* (34.4%), and *Staphylococcus epidermidis* (6.8%); while the anaerobic flora was dominated by *Bifidobacterium* sp. (13.4%) and *Clostridium bifermentans* (3.4%) [70].

Taken together, the results of the abovementioned studies on Western as well as Indian infants suggest that the overall pattern of colonization of the intestinal microflora during infancy remains broadly similar, with a large number of genera common to both populations. However, differences exist with respect to the specific species detected, their relative proportions, and the exact sequence of colonization. The main results of the Indian studies discussed here have been summarized in Table 2.

## 3. Conclusion

The beneficial effects of gut microflora have been known for a long time, as has been the fact that the infant gut microflora is different from that of adults. The gut commensals not only aid in nutrient metabolism but also play a vital role in providing immunity against pathogenic microbes. Hence, a healthy gut microflora is vital for the health of the infant. In this review, we analyzed the available literature on the development and characteristics of the infant gut microflora in Western infants and performed a comparative analysis with the limited data available on Indian infants. From our analysis, it appears that the gut microflora of infants from the two populations is more similar than expected. The temporal sequence of colonization remains the same, with aerotolerant and facultative anaerobes colonizing the gut first and paving the way for the growth of a predominantly anaerobic microflora later on. Several bacterial genera are common between the two populations. In particular, the presence of bifidobacteria and lactobacilli in infants in both populations, specifically in breastfed infants, has important clinical implications given that most probiotic formulations contain these two bacterial genera and most prebiotics are also formulated to stimulate their growth. More studies investigating the development and composition of the gut microflora in Indian infants, as well as comparative studies between Indian and Western infants, are warranted to gain further insight into this issue.

## Figures and Tables

**Table 1:** Evolution of the gut microflora in Western breastfed infants [1, 14, 55, 57, 58].

Phase of infancy		Family/genus/species	Phylum	Aerobic/anaerobic
Initial phase of bacterial acquisition (first 1-2 weeks after birth)	Immediately after birth	*Escherichia coli*	Proteobacteria	Facultative anaerobe
		*Streptococcus*	Firmicutes	Facultative anaerobe
	Subsequently	*Staphylococcus*	Firmicutes	Facultative anaerobe
		*Enterococcus*	Firmicutes	Facultative anaerobe
		*Lactobacillus*	Firmicutes	Facultative anaerobe/microaerophile
	Postnatal day 4-7	*Bacteroides*	Bacteroidetes	Obligate anaerobe
		*Bifidobacterium*	Actinobacteria	Anaerobe
		*Clostridium*	Firmicutes	Obligate anaerobe

Remaining period of exclusive breastfeeding (up to approximately 4 months of age)		*Bifidobacterium* (predominant)	Actinobacteria	Anaerobe
		*E*. *coli* (decrease in proportion)	Proteobacteria	Facultative anaerobe
		*Streptococcus* (decrease in proportion)	Firmicutes	Facultative anaerobe
		*Bacteroides* (decrease in proportion)	Bacteroidetes	Obligate anaerobe
		*Clostridium* (decrease in proportion)	Firmicutes	Obligate anaerobe

From introduction of solid food (4-6 months of age) to cessation of breastfeeding (around 1 year of age)		Enterobacteriaceae	Proteobacteria	Facultative anaerobe
		*Enterococcus*	Firmicutes	Facultative anaerobe
		*Bacteroides*	Bacteroidetes	Obligate anaerobe
		*Clostridium*	Firmicutes	Obligate anaerobe
		Anaerobic *Streptococcus*	Firmicutes	Anaerobe
		*Bifidobacterium*	Actinobacteria	Anaerobe
		*Lactobacillus*	Firmicutes	Facultative anaerobe/microaerophile

After completion of weaning (approximately 2 years of age)		Gut microflora begins to resemble that of adults		

**Table 2:** Summary of studies investigating the gut microflora in Indian infants.

Authors	Year	Study population	Location	Main findings
Albert et al. [59]	1977	South Indian infants aged 1-20 months	Vellore, Tamil Nadu	(i) The normal gut microflora was predominantly anaerobic; bifidobacteria were the most abundant microbes (ii) Aerobic bacteria mainly comprised enterobacteria and enterococci

Balamurugan et al. [61]	2008	South Indian children aged 2-3 years	Vellore, Tamil Nadu	(i) Bifidobacteria were the most abundant gut bacteria (ii) *Lactobacillus acidophilus* was also detected (iii) Both bacteria decreased in abundance after the age of 2-3 years

Balamurugan et al. [60]	2010	Term-born neonates in a tertiary care hospital in southern India	Vellore, Tamil Nadu	Enterobacteria (*Escherichia coli*) and bifidobacteria (*Bifidobacterium longum* subspecies *infantis*) were the predominant gut bacteria

Pandey et al. [63]	2012	Full-term, breastfed infants	Pune, Maharashtra	(i) Gut microflora of vaginally born infants on postnatal day 7 was dominated by *Acinetobacter* sp. (44%), followed by *Bifidobacterium* sp. and *Staphylococcus* sp. (ii) In cesarean-born infants, *Citrobacter* sp., *E. coli*, and *Clostridium difficile* were the dominant microbes; bifidobacteria were absent

Sharma et al. [70]	2012	Full-term infants around 4 weeks of age	New Delhi	(i) Predominant aerobic organisms: *Micrococcus* sp. (3.4%) (ii) Predominant facultative anaerobes: *Klebsiella pneumoniae* (41.3%), *Enterococcus faecium* (34.4%), *E. coli* (24.1%), *Proteus* sp. (10.3%), and *Staphylococcus epidermidis* (6.8%) (iii) Predominant anaerobic organisms: *Bifidobacterium* sp. (13.4%) and *Clostridium bifermentans* (3.4%)

Kabeerdoss et al. [62]	2013	Term-born infants in a south Indian hospital followed up from birth till 6 months of age	Vellore, Tamil Nadu	(i) Enterobacteria and lactobacilli were the predominant gut bacteria in the first 2 days of life (ii) Bifidobacteria and staphylococci increased by the fourth day (iii) *Bacteroides-Prevotella* group increased by around 6 months of age (iv) Enterococci were less abundant

Dinh et al. [64]	2016	Persistently stunted (cases) and normal children (controls) from a birth cohort in a south Indian slum community followed up from birth till 2 years of age	Vellore, Tamil Nadu	(i) Firmicutes (38.6%) and Proteobacteria (25.89%) were the most abundant bacteria in the overall cohort, followed by Actinobacteria (17.5%), Bacteroidetes (13.8%), and Verrucomicrobia (ii) Microflora of control children was enriched in the probiotic species *Bifidobacterium longum* and *Lactobacillus mucosae*

Chandel et al. [67]	2017	Full-term, cesarean-born infants	Odisha	(i) Firmicutes (28%; *Enterococcus*, *Clostridium*, and *Staphylococcus*) and Proteobacteria (64%; *Escherichia*, *Shigella*) were the predominant phyla on postnatal day 7 (ii) By postnatal day 60, the proportion of Proteobacteria reduced to 13%, and Firmicutes (49%; e.g., *Streptococcus*), Bacteroidetes (36%), and Actinobacteria (*Bifidobacterium*) increased

Attri et al. [68]	2018	Vaginally born, exclusively breastfed, full-term infants	Solan and Shimla, Himachal Pradesh	(i) First and second months of life: facultative anaerobic bacteria (Firmicutes and Proteobacteria) predominated (ii) Third and fourth months: Obligate anaerobes (Bacteroidetes and Actinobacteria) increased (iii) Clostridia were predominant throughout months 1-4 (20-30%) (iv) *Bifidobacterium adolescentis*, *B. bifidum*, *B. longum*, *B. pseudolongum*, and *B. breve* were also detected

Attri et al. [69]	2018	Full-term, vaginally born, exclusively breastfed infants	Solan, Himachal Pradesh	(i) In the first 4 months of life, *Enterococcus*, *Streptococcus*, and *Lactobacillus* were the predominant lactic acid-producing bacteria (ii) *B*. *breve* was the predominant species of bifidobacteria (iii) The diversity of lactic acid-producing bacteria and bifidobacteria increased over 4 months
